# Postpartum depression and infant feeding practices in a low income urban settlement in Nairobi-Kenya

**DOI:** 10.1186/s13104-016-2307-9

**Published:** 2016-12-08

**Authors:** Beatrice A. Madeghe, Violet N. Kimani, Ann Vander Stoep, Semret Nicodimos, Manasi Kumar

**Affiliations:** 1 School of Public Health, College of Health Sciences, University of Nairobi, Nairobi, 00100 Kenya; 2 Public Health, School of Public Health, College of Health Sciences, University of Nairobi, Nairobi, 00100 Kenya; 3 Psychiatry & Behavioral Sciences and Epidemiology, University of Washington, Child Health Institute, 6200 NE 74th Street, Suite 210, Seattle, WA 88115-1538 USA; 4 Clinical Research Associate, University of Washington, Child Health Institute, 6200 NE 74th Street, Suite 210, Seattle, WA 88115-1538 USA; 5 Department of Psychiatry, College of Health Sciences, University of Nairobi, Nairobi, 00100 (47074) Kenya

**Keywords:** Postpartum depression, Breastfeeding practices, Nutritional status, Kenya

## Abstract

**Background:**

Postpartum depression can compromise caregiving activities, including infant feeding practices, resulting in child malnutrition. The purpose of this study was to examine the effects of postpartum depression on infant feeding practices and malnutrition among women in an urban low income settlement in Nairobi-Kenya. We conducted a cross-sectional study based in Kariobangi North Health Centre in Nairobi County. The study sample included 200 mother-infant pairs visiting the Maternal and Child Health clinics for infant immunization at 6–14 weeks postpartum. We assessed postpartum depression using the Edinburgh Postpartum Depression Scale. Infant feeding practices were assessed based on World Health Organization infant and young child feeding guidelines. Nutritional status (weight for age) was ascertained using infants’ growth monitoring card (percentiles and z-score). We conducted logistic regression analyses to determine the relative odds of non-exclusive breast feeding and infant underweight among mothers with postpartum depression.

**Results:**

The prevalence of PPD was 13.0% (95% CI 8.3–17.7%). Taking into account differences in socioeconomic status of depressed and non-depressed mothers, non-depressed mothers had a 6.14 (95% CI 2.45–13.36) times higher odds of practicing exclusive breastfeeding than mothers who were depressed. Mothers with PPD had a 4.40 (95% CI 1.91–11.93) time higher odds of having an underweight infant than mothers without depression.

**Conclusions:**

This study contributes towards filling the knowledge gap regarding the adverse effects of postpartum depression on infant health in sub-Saharan Africa. We recommend more research on PPD using longitudinal designs to establish temporal ordering of these important public health problems and development of community-based interventions to address post-partum depression.

**Electronic supplementary material:**

The online version of this article (doi:10.1186/s13104-016-2307-9) contains supplementary material, which is available to authorized users.

## Background

Postpartum depression (PPD) is a mood disorder affecting 10–15% of women within a year of childbirth worldwide [1–6]. PPD can compromise care-giving activities and performance of parenting roles [7, 8]. PPD can affect infant feeding, with studies reporting that depressed mothers have increased odds of early termination of breastfeeding, and inappropriate feeding practices [9–13]. Several studies have also found a link between maternal depression and poor infant growth [14–17]. In a systematic review conducted in developing countries, mothers with depressive symptoms were 40% more likely to have underweight or height-stunted children than mothers who were not depressed [14, 18, 19]. The underlying connection between maternal depression and child malnutrition is not well etched out we say this as the population based studies from Ethiopia and South Africa [6, 20, 21] have found no association between maternal depression and malnutrition whereas clinic based studies tend to find associations between PND and infant growth [5, 7, 8, 15, 16, 18, 20, 22, 23]. If mothers are depressed they cannot feed their babies well, therefore, depression in mothers could adversely impact upon infant nutritional status, because nurturing and feeding an infant requires, mother’s full concentration and own well-being in place.

In Kenya malnutrition remains a leading cause of death for young children below age five with around 35% percent of children under 5 years stunted, 16% being underweight, and 7% classified as wasted [24]. The purpose of this study was to investigate the association between postpartum depression, infant feeding practices and child malnutrition in Kenya. We chose as our study population women living in a low-income urban settlement in Nairobi where the prevalence of child malnutrition was known to be high [25, 26]. Depressed mothers in the early postpartum period may be at a higher risk for negative infant-feeding outcomes including decreased breastfeeding duration and increased breastfeeding difficulties [9, 20, 21, 23, 27]. These findings help posit that Kenyan mothers with PPD would be significantly less likely than mothers without depression to practice exclusive breastfeeding and would be significantly more likely to have infants who are underweight.

## Methods

### Study design

The study was conducted in Kenya-Nairobi City County at Kariobangi North-Health Centre. Ethical approval was obtained from The Kenyatta National Hospital/University of Nairobi Ethical and Research Committee (KNH/UoN-ERC Ref. P141/03/2014). All women gave written informed consent for themselves and their infants in Kiswahili prior to their inclusion in the study. Women with high score on EPDS or suicidal ideation or those presenting with mental health symptoms during the interview were referred for a follow up at a specialist mental health clinic in Mathare North. However the challenge in the system is often around follow-up as the social services system is neither too resourceful nor efficient.

### Setting

Kariobangi is located approximately 15 km northeast of Nairobi city centre. It is a low-income residential estate that consists of both lower middle class and informal settlement. The informal settlement is characterized by high unemployment rate, poor housing, and poor public services such as sanitation, clean and water. Kariobangi North-Health Centre is the only public health center that provides services for the entire Kariobangi North area together with other few private dispensaries that offer services in this area. The facility offers curative outpatient services, a maternal and child health clinic with antenatal and postnatal care, family planning, growth monitoring and promotion, immunizations, Antiretroviral Therapy, and HIV counseling and testing. The maternal and child health department receives around 50–60 patients per day making 1500–1800 patiently monthly. Poor children living in urban areas remain disadvantaged, in 2012 an estimated under-five mortality rate in slums of Nairobi was 79.8 per 1000 compared to the 2009 estimate of 63.4 per 1000 [24, 26]. A large proportion of slum dwellers are adolescents and young adults from different tribal and cultural backgrounds who are between ages 15–45. The source of livelihood mainly comes from informal income-generating activities such a retail trade of consumable goods in small kiosks or paid casual work.

### Study participants

Women were eligible for recruitment if they were 17–44 years of age with 6–16 weeks-old infants and attended the maternal and child health (MCH) clinic for infant immunization visits. Women with pre-partum cases of serious mental illness, as evidenced by documentation of diagnoses or psychotropic medication prescription were excluded as these could be a distinct group themselves contributing to more bias or greater heterogeneity. Kariobangi is a low-income residential estate in north eastern part of Nairobi, Kenya. It consists of both lower middle class and slum-type dwellings and split into two parts: Kariobangi North and Kariobangi South. A consecutive sampling method was used where every eligible mother-infant pair was recruited who attended the 6th and 16th week immunization clinics on active MCH clinic days between July 14 and August 15, 2014 and met the inclusion criteria. Our estimated sample size was 216 women and we were able to successfully recruit the number needed. All women who were eligible and invited to the study agreed to participate as the lead researcher well-integrated her work as part of the everyday MCH clinic functioning and offered extra hands and nutritional support however we had 7.4% N = 16 missing data of mother-infant pair, the missing data include information such as mothers age, income level, infant’s age etc., at times the clinic was bustling with women and there were oversights in keeping a tab on the mothers moving from one clinic to the next. Women who were not residing in the Kariobangi area were excluded. We did not screen for any preexisting illnesses or probe about HIV status and do recognize that these are limitations of our work. We tried as much as possible to make data collection efficacious, not difficult for mothers to respond to and one with least interruption to the flow of the clinic.

### Data collection procedure and measures

The nurse in charge of the MCH clinic approached eligible mothers who came to the clinic and invited them to participate in the study. After registering their attendance with the nurse, mothers were approached for participation, the study purpose was explained to them and sought their consent and interest in participating, and after consent form signed the study tools were administered.

The lead author, who is a nutritionist and a public health researcher, conducted interviews with participating mothers in one section of the clinic. Mothers filled in a socio-demographic questionnaire which probed their age, marital, educational and occupational status including monthly income, dwelling type and the number of children. Postpartum depression was assessed using Edinburgh Postnatal Depression Screening [28]. The EPDS includes 10 items focusing on mood in the postpartum period in the past one week; it has a clinical postnatal depression score cut-off of 13 and above. Women with high score on EPDS or suicidal ideation or those presenting with mental health symptoms during the interview were referred to the mental health clinic held every Tuesday of the week at the health center or refereed to a nearby hospital which offer mental health services.

**Fig. 1:**
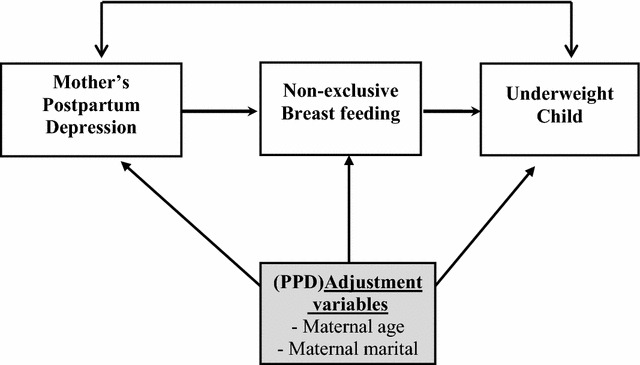
Theoretical model in which mediational role of non-exclusive breastfeeding in association between PPD and child malnutrition was tested

EPDS is a well-established instrument for postpartum depression that has demonstrated acceptable clinical utility as a screening scale in SSA [18, 29–31]. Members of the University of Nairobi Maternal and Child mental health working group had recently carried out a formal Kiswahili translation of EPDS including discussion of face validity issues and cross-cultural emic-etic issues in translation [32] and it was this version of the tool that was administered orally to the women. The lead researcher first gave the socio-demographic tool followed by EPDS and then gave the breastfeeding questionnaire which was followed by the nutritional status. Infant feeding practices were determined via an interviewer-administered, adapted and modified USAID Tool Kit for monitoring and evaluating breastfeeding practices [33]. Based on WHO infant and young children feeding recommendations for infants 0–6 months of age, feeding practices were classified as “exclusive breastfeeding” or “non-exclusive breastfeeding” on the basis of whether or not the infant had consumed solid foods or liquids other than milk with the exception of oral rehydration solution, drops or syrups consisting of vitamins, minerals supplements or medicines [34] (Fig. 1).

The first author used anthropometric measurements to assess infant nutritional status. The weights for babies were taken using a weighing scale and interpreted through infant growth monitoring card as percentiles and *z*-scores. In the MCH clinic, a trained nurse or (sometimes this could be a nutritionist) was responsible in taking the weight following the WHO guidelines of taking height and weight. In this study, the focus was on weight for age “underweight only”. Weight-for-age is a composite index of height-for- age and weight-for-height. It takes into account both acute and chronic malnutrition. The birth weights, weight at six weeks were used along with weight at the point of assessments to rule out if the infants were underweight or growing well. All children in this study with weight for age of-1SD, -2 SD and -3SD were considered underweight. For the premature or underweight unborn babies the direction of the growth curve was more important than the position of the curve on the chart from birth weight to the age of the assessment. A flat growth curve or a downward growth curve indicated danger which could indicate malnutrition. We do recognize that in focusing only on underweight categories we were not able to differentiate between stunting or wasting or a combination of both. It would have been interesting to know whether underweight was due to stunting, wasting or both.

### Statistical methods

Sample size was determined based on World Health Organization estimate prevalence of depressive symptoms amongst women in developing countries at anywhere from 15% onwards [14]. Minimum required sample size was calculated using the prevalence formula by [35]. Pearson’s Chi square tests were used to evaluate associations between predictor and outcome variables. Using regression, we created a model with both feeding practices and PPD as potential predictors of infant nutritional status to explore the possibility that feeding practices explained or mediated part of the association between PPD and the infant nutritional status outcome. In this study analyses p < 0.05 was considered statistically significant. SPSS version 20 was used for analysis.

## Results

### Social demographic characteristics of mothers

Of 216 women who were recruited, 200 mother-infant pairs ‘complete data was entered and included in the analysis. As shown in Table 1, women’s age ranged from 17 to 39 years. Eighty-seven percent were married. Nearly two-thirds had had some primary school education but had not advanced to secondary school, from the total sample, 59% were not earning or formally employed and 57% were living in semi-permanent dwellings. For over 75% of the participants, the family income was between Ksh 5000 and 10,000/month (that is around 60–120 USD). See Table 1 for compilation of socio-demographic factors associated with postpartum depression, malnutrition status and infant feeding practices.

**Table 1:** Description of study sample

Demographic variables	N	%
Maternal age		
Less than 20 years	19	9.5%
20–25 years	81	40.5%
25 years and up	100	50.0%
Marital status		
Married	174	87.0%
Single/divorced/separated/widowed	26	13.0%
Maternal education		
None	5	2.5%
Primary only	128	64.0%
Secondary only	64	32.0%
Tertiary only	3	1.5%
Occupation		
Housewife/stay at home	118	59.0%
Employed	15	7.5%
Has business	52	26.0%
Other	15	7.5%
Monthly income		
Less than 5000 Ksh	37	18.5%
5000–10,000 Ksh	157	78.5%
Above 10,000 Ksh	6	3.0%
Living situation		
Temporary	79	39.5%
Semi-temporary	114	57.0%
Permanent	7	3.5%
Infant nutrition classification		
Normal	131	65.5%
Mild malnutrition	41	20.5%
Moderate malnutrition	28	14.0%
Breast feeding practice		
Exclusive breast-feeding	146	73.0%
Non-exclusive breast-feeding	54	27.0%
EPDS Score		
Mean, SD	6.23	4.92
Infant age in months		
Mean, SD	1.01	0.71

### Prevalence of postpartum depression

Of the 200 mothers, 26 (13.0%; 95% CI 8.3–17.7%) were found to have postpartum depressive illness, as indicated by an EPDS score of ≥13. Socio-demographic factors that reflected the mother’s level of economic security were associated with postpartum depression (see Table 2). These findings indicate that mothers characterized by low socioeconomic status were significantly more likely than other mothers to be depressed. The prevalence of PPD amongst single mothers was 34.6% (95% CI 15.0–54.2%), among mothers with a family income level of less than Ksh 5000/month it was 27.0% (95% CI = 12.0–42.0%), and among mothers whose occupation was classified as “other” was 40.0% (95% CI 11.9–68.1%). The ‘other’ category meant any irregularly organized activities such as cleaning houses, doing laundry for other households where one works as a casual laborers but are neither formally employed or run small business owners.

**Table 2:** Socio-demographic profile across three study variables- post-partum depression, infant weight, and breast feeding practices

Demographic variables	Postpartum depression					Infant weight					Breast feeding practices				
	With (N = 26)		Without (N = 174)		p	Underweight (N = 69)		Normal (N = 131)		p	Non-exclusively (N = 54)		Exclusively (N = 146)		p
	N	%	N	%		N	%	N	%		N	%	N	%	
Maternal age															
Less than 20 years	4	15.4	17	9.8	0.428	9	13.0	12	9.2	0.548	6	11.1	15	10.3	0.873
20–25 years	12	46.2	68	39.1		29	42.0	51	38.9		20	37.0	60	41.1	
25 years and up	10	38.5	89	51.2		31	44.9	68	51.9		28	51.9	71	48.6	
Marital status															
Married	17	65.4	157	90.2	0.000	55	79.7	119	90.8	0.026	41	75.9	133	91.1	0.005
Single/divorced/separated/widowed	9	34.6	17	9.8		14	20.3	12	9.2		13	24.1	13	8.9	
Maternal education															
None	2	7.7	3	1.7	0.270	3	4.4	2	1.5	0.529	2	3.7	3	2.1	0.661
Primary only	17	65.4	111	63.8		46	66.7	82	62.6		34	63.0	94	64.4	
Secondary only	7	26.9	57	32.8		19	27.5	45	34.4		18	33.3	46	31.5	
Tertiary only	0	0.0	3	1.7		1	1.5	2	1.5		0	0.0	3	2.1	
Occupation															
Housewife/stay at home	16	61.5	102	58.6	0.004	40	58.0	78	59.5	0.287	28	51.9	90	61.6	0.026
Employed	0	0.0	15	8.6		3	4.4	12	9.2		3	5.6	12	8.2	
Has business	4	15.4	48	27.6		18	26.1	34	26.0		14	25.9	38	26.0	
Other	6	23.1	9	5.2		8	11.6	7	5.3		9	16.7	6	4.1	
Monthly income															
Less than 5000 Ksh	10	38.5	27	15.5	0.015	13	18.8	24	18.3	0.647	15	27.8	22	15.1	0.111
5000–10,000 Ksh	16	61.5	141	81.0		55	79.7	102	77.9		38	70.4	119	81.5	
Above 10,000 Ksh	0	0.0	6	3.5		1	1.5	5	3.8		1	1.9	5	3.4	
Living situation															
Temporary	15	57.7	64	36.8	0.096	32	46.4	47	35.9	0.228	27	50.0	52	35.6	0.161
Semi-temporary	11	42.3	103	59.2		36	52.2	78	59.5		26	48.2	88	60.3	
Permanent	0	0.0	7	4.0		1	1.5	6	4.6		1	1.9	6	4.1	

### Feeding practices

When queried about their feeding practices, 27% of mothers had supplemented their infants particularly with water (31%), formula (8%), cow milk (6%), and soft porridge (17%). On further probing, many women reported that their children were not getting satisfied with the breast milk alone. Others described low volumes of breast milk due to lack of food while others reported that their children suffered digestive complications like constipation prompting them to give water. Socio-demographic factors such as marital age, marital status, parity and income were identified a priori to be adjustment variables in the association between non-exclusive breastfeeding and PPD. Women with post-partum depression were significantly more likely than women without PPD to have practiced non-exclusive breastfeeding (UOR 6.99, 95% CI 2.88–16.96; P < 0.001).

### Infant’s nutritional status

Using the weight-for-age indicator of malnutrition, 34% (95% CI 27.9–41.1%) of infants were determined to be underweight. As mentioned before socio-demographic factors were identified a priori to be adjustment variables in the association between infant weight and post-partum (these included marital age, marital status, parity and income). Infants of women with PPD were significantly more likely than infants of women without PPD to be underweight [OR 5.43; 95% CI (2.22–13.27); (P = 0.001)].

### Results of multivariate analyses

Associations between PPD and infant feeding practice and PPD and infant nutritional status remained strong and significant after adjusting for socio-demographic characteristics that were identified a priori. As shown in Table 3, odds ratios were only slightly diminished after adjusting for these potential confounders. The adjusted excess odds of non-exclusive breast feeding among mothers with PPD was 5.91 (95% CI 2.29–15.27) and of infant underweight status was 5.79 (95% CI 2.14–15.62). Findings from the exploratory evaluation showed that ~17% of the association between PPD and infant malnourishment might be explained by non-exclusive breast feeding.

**Table 3:** Logistic regression results for nutritional status of infants (underweight) and maternal practice of non-exclusive breast-feeding

Independent variables	Unadjusted		Adjusted^a^	
	OR	95% CI	OR	95% CI
Underweight	5.43	2.22–13.27	5.79	2.14–15.62
Non-exclusive feeding	6.99	2.88–16.96	5.91	2.29–15.27

^a^Adjusted for maternal age (continuous), marital status, parity and income

## Discussion

### Prevalence of postpartum depression

Our study found a 13% prevalence of post-partum depressive symptoms among women attending MCH clinic based in an urban settlement of Nairobi. This estimate lies within the wide range of prior PPD prevalence estimates (6.1–30.6%), reported amongst African mothers [1, 3, 31, 36]. A few studies conducted in South Africa found higher rates of PPD, for example Tomlinson and colleagues estimated prevalence of postpartum depression as 34.7% in a peri-urban settlement in Cape Town [37]. Explaining the differences among these prevalence estimates could be methodological differences in the ways the studies were conducted, the settings where the studies were carried out, timing of post-partum assessment, screening instruments used and cutoff values used to classify mothers as depressed [5, 18, 31]. As is apparent the EPDS is the most widely used PPD measure, but a range of cut-off points have been set, ranging from 10 to 13. In this study the prevalence estimate would have been 21%, (95% CI 15.3–26.7%) had the clinical cutoff had been lowered to a score of 10.

### Associations between PPD and socio-demographics characteristics

In this study postpartum depression was significantly associated with low socio- economic status where women with low levels of income were more likely to be depressed than mothers who had higher income. Marital status was also strongly associated with depression. Single mothers living in low-income urban settlements lack economic and child-rearing support from husbands and their extended families, thereby struggling more with life stress and consequently depression. Our findings are consistent with several studies that have reported how among mothers from lower socio-economic status, especially in the context of poor social support, are at increased risk of parenting difficulties and adverse child outcomes [15, 16, 22, 23, 36, 38].

### Infant feeding practices and postpartum depression

Our study found that women with PPD were more likely to introduce supplementary foods earlier than women who were not depressed. These results compare well to the results reported by [38] in Brazil who evaluated the association between postpartum depression and interruption of exclusive breastfeeding in the first 2 months of life and found that children of mothers with postpartum depressive symptoms were at higher risk of early interruption of exclusive breastfeeding. In Australia [27] reported early cessation of breastfeeding to be significantly associated with postnatal depression. In Nigeria, [23] reported that depressed mothers were more likely to stop breastfeeding earlier, and their infants were more likely to have episodes of diarrhea and other infectious illnesses. In our study two social determinants associated with non-exclusive breastfeeding included income level and marital status.

### Infant’s nutritional status and postpartum depression

This study provides further evidence of an association between postpartum depression and poor infant nutritional status. Low income Kenyan mothers living in urban settlements struggle to obtain enough food. When they have insufficient breast milk to nourish their infants, they supplement their infants’ diets with water and other foods, that then exposes infants to infectious diseases such as diarrhea, and, hence, their becoming become underweight [15, 39]. Our study findings suggest that post-partum depression may contribute to this vicious cycle. WHO recommends that mothers in LMIC settings exclusively breastfeed infants for the first six months to achieve optimal growth and development [34]. In under-resourced settings, where sanitation and safe water are often lacking, breastfeeding can be life-saving. Breastfeeding protects against infectious diseases, especially gastrointestinal infections, which largely contribute to child morbidity and mortality in low income countries [39].

Our results linking PPD and poor infant nutritional status are consistent with findings of previous studies. In Nigeria [23] showed that infants of depressed mothers had significantly poorer growth than infants of non-depressed mothers at the 3rd month. In Zambia, [16] found adverse infant health outcomes were proportionately greater in infants of depressed mothers and the associations with adjusted mean difference in infant weight and length too were statistically significant. Other independent associations with episodes of diarrhea, maternal education, infant age, supplementary feeding) and incomplete vaccination were identified as risk factors. Similarly in Malawi, [22] reported that mean length-for-age *z*-score for infants of mothers with PPD was significantly lower than for infants of mothers without PPD. The multivariate results show that maternal income, marital status, and employment status do not explain away the strong associations between maternal depression and infant nutritional outcomes.

## Study limitations

The cross-sectional study design hampers our ability to understand the temporal sequence and reciprocal associations amongst the health and behavioral issues of interest. It is plausible, for example, that babies who are low birth weight are difficult to breast-feed and that the frustration and stress of caring for a compromised infant contributes adversely to the mother’s emotional health. A longitudinal study design, besides establishing temporal sequence, could better evaluate potential mechanisms through which mother’s depression affects infant nutritional status. While this study is an important first step in shedding light on key public health concerns in Kenya, we are cautious in generalizing too broadly from the Kariobangi MCH clinic sample. Women who attended the MCH clinic to have their infants immunized may not have represented all new mothers in the Kariobangi area. We may have seen a higher prevalence of depression and different strengths of associations between PPD and infant nutritional outcomes had the study included all Kariobangi mothers who had given birth over the same time period.

Besides the fact that this was a cross-sectional study, our study lacks information that would have strengthened our ability to draw conclusions about associations between PPD and infant outcomes. Since we do not know about pre-partum depression status of mothers, we cannot sort out effects of post-partum depression from more long-standing depression. Knowing infant birthweight would also have added an important dimension to the study analyses.

## Conclusions

Our study found a strong cross-sectional association between postpartum depression, infant feeding practices, and child malnutrition, making a modest contribution to the knowledge base that sheds light on the links between the well-being of mothers and the health of their infants. It emphasizes providing mothers and families the support they need to carry out their crucial roles and it explicitly defines the obligations and responsibilities in this regard of governments and other concerned parties to help out as far as the Global Strategy for Infant and Young Child Feeding [34]. Maternal depression is one of the most well-documented risk factors for child and adolescent depression, The potential adverse effects of postpartum depression on the parenting practices and child physical and emotional development reinforce the need for programs aimed at prevention, early identification and effective treatment of PPD. To do so requires a paradigm shift—moving from a focus on individual treatment to a prevention approach that engages the entire family as the unit of care. Given the high prevalence of depression among women of childbearing age, practitioners working with women in prenatal care settings should routinely screen for depression and provide targeted treatment and preventive services. Clinical trials of Interpersonal Therapy show promise for group-based treatment of adults delivered by community health workers in E. Africa, [40] and for treatment of pregnant women living in low income settings in the U.S. [41]. Currently, an IPT trial with women who are HIV+ and depressed is underway in western Kenya [42]. We recommend more health resources targeting PPD and more research using community-based samples in low-income settings and longitudinal study designs with multiple assessments over the post-partum period to assess temporal timing and reciprocity between maternal mental health, feeding practices, and child growth and development. Such studies will enable us to target and design effective programming to address major global public health problems.

## Additional file

**Additional file 1.** Dataset of postpartum depression, infant feeding and child nutritional status study.

## Abbreviations

APHRC: African Population and Health Research Center
EPDS: Edinburgh Postnatal Depression Scale
KDHS: Kenyan Demographic and Health Survey
KNH/UoN-ERC: Kenyatta National Hospital/University of Nairobi Ethical and Research Committee
MCH: Maternal and Child Health
PPD: post partum depression
SSA: sub-Saharan Africa
SPSS: Statistical Package for Social Science
OR: odds ratio
CI: confidence intervals
USAID: United State Agency for International Development
WHO: World Health Organization
